# Antioxidant and Larvicidal Activity of Areal Parts of *Scrophularia striata* against Malaria Vector *Anopheles stephensi*

**Published:** 2018-06-13

**Authors:** Fatemeh Yousefbeyk, Hassan Vatandoost, Fereshteh Golfakhrabadi, Zahra Mirzaee, Mohammad Reza Abai, Gholamreza Amin, Mahnaz Khanavi

**Affiliations:** 1 Department of Pharmacognosy, School of Pharmacy, Guilan University of Medical Sciences, Rasht, Iran; 2 Department of Medical Entomology and Vector Control, School of Public Health, Tehran University of Medical Sciences, Tehran, Iran; 3 Department of Pharmacognosy, School of Pharmacy and Persian Medicine and Pharmacy Research Center, Tehran University of Medical Sciences, Tehran, Iran; 4 Faculty of Land and Food Systems University of British Columbia, British Columbia, Canada

**Keywords:** *Scrophularia striata*, Antioxidant activity, Larvicidal activity, *Anopheles stephensi*

## Abstract

**Background:**

*Scrophularia striata* is a perennial plant which is native in all parts of Iran, Turkey, and Azerbaijan. In this study, the total phenol content, antioxidant and larvicidal activities of total extract and different fractions of this plant were evaluated.

**Methods:**

The aerial parts of *S. striata* were collected from Boli village, Illam Province, western Iran in Apr 2013. The total phenol content of total extract and different fractions were evaluated by Folin-Ciocalteu method. Moreover, antioxidant activity was tested by DPPH and FRAPS assays. Larvicidal activity was investigated according to standard method described by WHO.

**Results:**

Ethyl acetate fraction (EF) had the highest content of total phenol (75.9±0.06mg Gallic acid equivalent/g dry extract). Furthermore, among the tested extract, methanol-water fraction (MWF), total methanol extract (TME) and water fraction (WF) showed the highest antioxidant activity in the DPPH assay (IC_50_= 226.8, 283.66 and 299.4 μg.ml^−1^, respectively). In FRAP assay MWF and WF and TME had the highest antioxidant activities (664.4±0.002, 565.3±0.003, 519.5±0.003mmol FeII/g dry extract, respectively). Ethyl acetate fraction had maximum larvicidal activity (LC_50_ 49.1ppm) followed by TME (LC_50_ 64.26ppm) and hexane fraction (HF) (LC_50_ 89.69).

**Conclusion:**

*Scrophularia striata* collected from west of Iran illustrated considerable antioxidant and larvicidal effects and further in vitro and in vivo experimental models for investigation would be required.

## Introduction

According to the word malaria report, in 2014, 198 million cases of malaria occurred globally in 2013 and led to 584000 deaths (1–3). In southern parts of Iran including Sistan and Baluchistan, Hormozgan and some parts of Kerman provinces, this disease remains as a vital public health problem (4). Six *Anopheles* vectors exist in this area including *An. culicifacies*, *An. stephensi*, *An. dthali*, *An. fluviatilis*, *An. superpictus* and *An. pulcherrimus* (5). The present proliferation of malaria is due basically to increasing resistance of mosquitoes to current insecticides (6).

Plant materials have been investigated for their susceptibility in controlling the malaria vector due to their ovicidal, larvicidal and adulticidal activities. The most susceptible stage to attack mosquitoes is larval stage as they are concentrated in smaller areas. Therefore, interrupting mosquito life cycle at larval stage is one of the important methods for controlling malaria transmission (7).

The genus *Scrophularia* (Scrophulariaceae) comprises about 300 known species. The various species of *Scrophularia* genus have been traditionally used in medical conditions including skin inflammatory disease like scabies, tumors, eczema (8), and psoriasis, inflammatory disorders fever, constipation, pharyngitis, neuritis, laryngitis affections (9). Among these species, *S. ningpoensis* Hemsl has been used for treatment of fever laryngitis, pharyngitis, neuritis and constipation swelling in China. Moreover, *S. grossheimi* and *S. nodosa* are used as diuretic agents (10). Recent studies reported antimalarial, antiprotozoal and antimycobacterial activities of *S. cryptophila* (11).

Phytochemical studies indicated the presence of saikosaponins, iridoids and phenylpropanoid glycosides such as angoroside A, angoroside C, angoroside D, acteoside and isoacteoside in *S. scorodonia* L. (8), iridoid glucosides including catalpol, 6-O-methylcatalpol and aucubin, phenylethanoid glycoside like angoroside C in *S. lepidota* (12), resin glycosides including crypthophilic acids A–C in *S. cryptophila* (11).

Many natural compounds have been reported for larvicidal activity. For example, flavonoids namely poncirin, rhoifolin, naringin and marmesin from *Poncirus trifoliate* (13), lupinfolin and rotenone and deguelin from *Derris trifoliate* (14), new iridoid glycosides like 6′-O-rhamnosylharpagide and 6-O-xylosylharpagoside-B form *Ajuga remota* (15) and coumarins such as umbelliferone, herniarin, psoralen and xanthotoxin from *Cnidium monnieri* (16) and coumarin derivative pachyrrhizine from *Neorautanenia mitis* (17).

*Scrophularia striata* Boiss is a perennial plant grown in all parts of Iran as well as Turkey and Azerbaijan. These species have been used to cure different inflammatory diseases such as allergy, rheumatics and chronic inflammatory disorders in Iranian folk medicine (18). So far, phytochemical investigation revealed the presence of cinnamic acid, flavonoids such as quercetine, isorhamnetin-3-O-rutinoside, and nepitrinandand, and one phenyl propanoid glycoside acteoside 1 in this plant (10). Studied showed the inhibitory effect of *S. striata* extract on matrix metalloproteinases and astrocyte cancer cell line (1321) (18, 19).

Different extracts from the aerial part of *S. canina* were evaluated for the insecticidal activity against larvae and adult females of *Culex pipiens molestus*. The most toxicity was revealed by the petroleum ether extract against second-instar larvae (48h, LC_50_= 23.5 ppm) and by the hexane extract against fourth-instar larvae (48h, LC_50_= 23.6ppm) (20). The leaves of *S. nodosa* showed larvicidal activity against *Cx. quinquefasciatus* (75.3% mortality) (21). The ethanol root extract of *S. lepidota* exhibited anti-protozoal activity (IC_50_ 40.6μg.ml^−1^) and plasmodial enoyl-ACP reductase (FabI) enzyme inhibitory activity (IC_50_ 100μg.ml^−1^), a key enzyme of fatty acid biosynthesis in *Plasmodium falciparum* (12).

In the present study, total extract and different fractions of *S. striata* were investigated for larvicidal properties against main malaria vector, *An. stephensi*. In addition, total phenol content and antioxidant activity (using DPPH and FRAP methods) of this plant were evaluated.

## Materials and Methods

### Plant material and extraction

The aerial parts of *S. striata* were collected from Boli village, Ilam Province, western Iran in Apr 2013. The voucher specimen was deposited in herbarium of faculty of Pharmacy, Tehran University of Medical Sciences (Herbarium number: 6748-TEH).

The aerial parts (500g) were aired dried in shade and fractionated with hexane, ethyl acetate, methanol, methanol-water (1:1) and water at room temperature, consequently. Total methanol extract was prepared from aerial parts by percolation method. All the extract and fractions were dried using rotary evaporator to give 2.8g, 6.5g, 60g, 48.2g, 7.7g and 28.7g residues from hexane, ethyl acetate, methanol, methanol-water (1: 1), water fractions and total extract, respectively.

## Antioxidant activity

### DPPH radical-scavenging activity assay

The antioxidant activity of extracts was investigated by the DPPH (2, 2′-diphenyl-1-picrylhydrazyl) free radical scavenging method according to an established protocol. Sample solutions (1ml) were prepared in methanol at different concentration. Then the solutions added to DPPH methanol solution (2ml, 40μg.ml^−1^) and incubated at room temperature for 30 min. The absorbance was measured at 517nm. Vitamin E and butyl hydroxyanisole (BHA) were used as positive controls. IC_50_ values (indicate the concentration of the test samples providing 50% radical scavenging) were calculated from graph-plotted scavenging percentage against extract concentration (22).

### Ferric reducing antioxidant potential (FRAP scavenging) assay

The FRAP assay was done by using the method described (23, 24). The FRAP reagent contained 5 ml of a (10mmol.l^−1^) TPTZ (2, 4, 6-tripyridyl-s-triazine) solution in 40 mmol.l^−1^ HCl plus 5ml of (20mmol.l^−1^) FeCl_3_ and 50ml of (0.3 mmol.l^−1^) acetate buffer, pH 3.6 was prepared freshly. Aliquots of each extract (50μl) were mixed with FRAP reagent (1.5ml). The mixtures were incubated at 37 °C, for 10min. Finally, the absorbance of each sample was measured at 593nm. For construction of calibration curve, five concentrations of FeSO_4_, 7H_2_O (125, 250, 500, 750, 1000 mmol.l^−1^) were prepared to used. The antioxidant activities were calculated as the concentration of antioxidants having a reducing ability equivalent for 1mmol.l^−1^ FeSO_4_ (22, 25).

### Determination of total phenolic contents

Total phenolic contents of extracts were determined by Folin-Ciocalteu method (26). Folin-Ciocalteu (Merk) reagent diluted to ten-fold with distilled water. Then 5ml of this solution was added to 1ml of each extract (1 mg.ml^−1^) and allowed to stand at room temperature for 10min. A 4ml sodium bicarbonate solution (75g.l^−1^) was added to the mixture. After 30min at room temperature, absorbance was measured at 765nm using a UV spectrophotometer (Pharmacia Biotech). Total phenolic contents were quantified by calibration curve obtained by measuring the absorbance of a known concentration of Gallic acid (GA) standard (20–200mg.l^−1^). The concentrations are expressed as milligrams of Gallic acid equivalents (GA) per gr dry extract (22, 27).

### Bioassays and larval mortality

Fourth instar larvae of *An. stephensi* Bandar-Abbas strain was exposed to test concentrations of 20, 40, 80, 160 and 320ppm of each extract (solvent: Ethanol) for 24h according to standard method described by WHO (1981).

Briefly, 1ml of appropriate dilution of each extract with 224ml of water and 25 larvae in 25ml water mixed and total volume was 250 ml (2). For control, only 1ml of ethanol with 224ml of water and 25 larvae in 25ml water mixed and total volume was 250ml. The experiment was repeated four times on different days. The percentage of mortality was reported from the average for the four replicates after 24h exposure period. From the regression line between logarithmic dose and probit mortality, the LC_50_ was measured (28, 29).

The investigation of larvicidal activity has been carried out in the insectarium of Department of Medical Entomology and Vector Control, School of Public Health, Tehran University of Medical Sciences, Tehran, Iran.

## Results

Ethyl acetate fraction (EF) had the highest content of total phenol (75.9±0.06mg Gallic acid equivalent/g dry extract) (Table 1). Among the tested extract, methanol-water fraction (MWF), total methanol extract (TME) and water fraction (WF) showed highest antioxidant activity in the DPPH assay (IC_50_= 226.8, 283.66 and 299.4μg.ml^−1^, respectively). In FRAP assay MWF and WF and TME had the highest antioxidant activities (664.4±0.002, 565.3±0.003, 519.5±0.003mmol FeII/g dry extract, respectively).

**Table 1.** Antioxidant activity and total phenolic content of total methanol extract and different fractions from aerial parts of *Scrophularia striata*

	**DPPH (IC_50_: μg.ml^−1^)**	**FRAP (mmol FeII/g dry extract)**	**Total phenol contents (mg GAE/g dry extract)**
**TME**	283.7	519.5±0.003	64.8±0.03
**HF**	>1000	248.8±0.035	**-**^a^
**EF**	505.2	444.28±002	75.9±0.06
**MF**	511.3	513.7±0.026	41.7±0.04
**MWF**	226.8	664.4±0.002	56.2±0.02
**WF**	299.4	565.3±0.003	33±0.06

Key to extracts employed: TME: Total methanol extract, HF: hexane fraction, EF: ethyl acetate fraction, MF: methanol fraction, MWF: methanol-water fraction, WF: water fraction.

a not detected.

The total extract and fractions of *S. striata* were effective against *An. stephensi* with LC_50_ ranged between 49.15 to 1265.96ppm. Ethyl acetate fraction had maximum larvicidal activity (LC_50_ 49.15ppm) followed by TME (LC_50_ 64.2623ppm) and HF (LC_50_ 89.69) (Table 2).

**Table 2.** Probit regression line parameters of total extract and different fractions of *Scrophularia striata* against *Anopheles stephensi*

	**Intercept**	**Slope ± SE**	**LC_50_ (ppm), 95%C.I.**	**LC_90_ (ppm), 95%C.I.**	**λ^2^ table (df)**	**P-value**
**TME**	−4.5092	2.4941±0.537	64.2623 (27.2325–131.1766)	209.8017 (109.8552–2827.5650)	24.405*	0.001
**HF**	−2.4518	1.2556±0.144	89.6911 (72.3973–112.2429)	940.7311 (577.0739–1990.1654)	13.345*	0.001
**EF**	−2.7150	1.6051±0.156	49.1497 (40.1770–58.6299)	309.0209 (231.3864–462.1751)	13.345*	0.001
**MF**	−4.6954	1.9658±0.199	244.6640 (203.2745–310.4233)	1097.8066 (747.3601–1921.5333)	13.345	0.001
**MWF**	−6.5143	2.0977±0.424	1265.9601 (607.7026–2645.3743)	5161.5548 (2523.4135–75009.0794)	13.345	0.001
**WF**	−7.9868	2.6359±0.195	1071.3972 (949.6278–1205.7662)	3282.1074 (2756.2862–4091.2804)	13.345	0.001

Key to extracts employed: TME: Total methanol extract, HF: hexane fraction, EF: ethyl acetate fraction, MF: methanol fraction, MWF: methanol water fraction, WF: water fraction, LC_50_ ±95% C.L= lethal dose cause 50% mortality, 95% confidence interval, LC_90_ ±95% C.I= lethal dose cause 90% mortality, 95% confidence interval, (df)= degree of freedom, P= P-value

## Discussion

*Scrophularia striata* is a perennial plant which is native in all parts of Iran as well as Turkey and Azerbaijan. In this study total methanol extract and different fractions from aerial parts of *S. striata* were investigated for antioxidant and larvicidal activities. According to our results, EF and TME had the highest amount of total phenolic compounds. *Scrophularia steriata*, as highest amount of total phenolic compounds, was reported in ethanol 70% and ethyl acetate extracts (79.7 and 65.5mg Gallic acid equivalent/g dry extract, respectively). Moreover, water extract of this plant was reported to have better radical scavenging activity (IC_50_ 195μg.ml^−1^) (30).

Furthermore, evaluation of larvicidal activity of *S. striata* revealed that LC_50_ value of total extract and fractions ranged between 49.15 to 1265.96ppm, and the EF had maximum larvicidal activity (LC_50_ 49.15ppm). Essential oils and extracts of different plant species have been investigated for larvicidal activity. For instance, the essential oil prepared from seeds of *Heracleum persicum* had larvicidal effect with LC_50_ value of 104.80ppm. The essential oil of *Cupressus arizonica* revealed significant larvicidal activity against *An. stephensi* with LC_50_ 79.30ppm (31). Moreover, for *Coriandrum sativum* LC_50_ value of 120.95ppm and for *Cymbopogon olivieri* 321.90ppm were reported (5), which their activities were low when compared with EF and TME of *S. striata*. The LC_50_ of essential oils from *Tagetes minuta* and *Foeniculum vulgare* were 1.05 and 20.10ppm, respectively (5), these plants were more effective than *S. striata.*

The methanol extract of *T. minuta* had better LC_50_ value (2.5ppm) followed by methanol and aqueous extract of *Nelumbo nucifera* (8.89 and 11.82ppm, respectively) and methanol extract of *Cassia fistula* (17.97ppm) when compared with the extract and fractions of *S. striata* (32–34).

Different extracts of fruits and leaves of *Centratherum anthelminticum* were tested for larvicidal activity against *An. stephensi* and the petroleum ether extract of fruits (LC_50_ 162.60) were more toxic than that of leaf extract (LC_50_ 522.94) (7). Petroleum ether extract from leaves of *Artemisia annua* had larvicidal activity with LC_50_ value of 263 ppm (7). The leaf and seed methanol extracts of *Clitoria ternatea* showed dose-dependent larvicidal activity against *An. stephensi* with LC_50_ values of 555.6 and 116.8ppm, respectively (35). The leaves petroleum ether extract of *Gymnema sylvestre* exhibited the highest mortality in the concentration of 1000ppm against the larvae of *An. subpictus* (LC_50_ 166.28 ppm) (36). Moreover, the leave methanol extract of *Calotropis gigantea* showed larvocidal activity (LC_50_ 121.69ppm) (37). Larvicidal activity of aceton extract of *Millingtonia hortensis* (LC_50_ 223.9ppm) and ethanol extract was from peels of *Citrus sinensis* (LC_50_ 291.69ppm) (35, 38).

Extracts of *A. annua*, *C. anthelminticum* (7), *Citrus sinensis*, *C. ternatea* (35), *C. gigantean* (37), *G. sylvestre* (36) and *M. hortensis* (38) were less active than EF and TME of *S. striata*.

## Conclusion

EF and TME of *S. striata* possesses better larvicidal activity against *An. stephensi* than other fractions. Moreover, antioxidant activity of MWF, WF, and TME were higher than other fractions in both DPPH and FRAP assays. Finally, complete phytochemical investigation is suggested to reveal most effective compound in this native species.
